# Altered Cellular Protein Quality Control System Modulates Cardiomyocyte Function in Volume Overload-Induced Hypertrophy

**DOI:** 10.3390/antiox11112210

**Published:** 2022-11-08

**Authors:** Kamilla Gömöri, Melissa Herwig, Roua Hassoun, Heidi Budde, Nusratul Mostafi, Simin Delalat, Suvasini Modi, Merima Begovic, Tamara Szabados, Judit Pipis, Nikolett Farkas-Morvay, István Leprán, Árpád Kovács, Andreas Mügge, Péter Ferdinandy, Anikó Görbe, Péter Bencsik, Nazha Hamdani

**Affiliations:** 1 Department of Cellular and Translational Physiology, Institute of Physiology, Ruhr University Bochum, 44801 Bochum, Germany; 2 Institut für Forschung und Lehre (IFL), Molecular and Experimental Cardiology, Ruhr University Bochum, 44801 Bochum, Germany; 3 Department of Cardiology, St. Josef-Hospital, Ruhr University Bochum, 44801 Bochum, Germany; 4 HCEMM-Cardiovascular Research Group, Department of Pharmacology and Pharmacotherapy, University of Budapest, HU-1089 Budapest, Hungary; 5 Department of Pharmacology and Pharmacotherapy, University of Szeged, HU-6720 Szeged, Hungary; 6 Pharmahungary Group, HU-6720 Szeged, Hungary; 7 Cardiovascular and Metabolic Research Group, Department of Pharmacology and Pharmacotherapy, Semmelweis University, HU-1089 Budapest, Hungary

**Keywords:** volume overload, hypertrophy, oxidative stress, heat shock proteins, protein quality control

## Abstract

Volume-induced hypertrophy is one of the risk factors for cardiac morbidity and mortality. In addition, mechanical and metabolic dysfunction, aging, and cellular redox balance are also contributing factors to the disease progression. In this study, we used volume overload (VO), which was induced by an aortocaval fistula in 2-month-old male Wistar rats, and sham-operated animals served as control. Functional parameters were measured by transthoracic echocardiography at termination 4- or 8-months after VO. The animals showed hypertrophic remodeling that was accompanied by mechanical dysfunction and increased cardiomyocyte stiffness. These alterations were reversible upon treatment with glutathione. Cardiomyocyte dysfunction was associated with elevated oxidative stress markers with unchanged inflammatory signaling pathways. In addition, we observed altered phosphorylation status of small heat shock proteins 27 and 70 and diminished protease expression caspases 3 compared to the matched control group, indicating an impaired protein quality control system. Such alterations might be attributed to the increased oxidative stress as anticipated from the enhanced titin oxidation, ubiquitination, and the elevation in oxidative stress markers. Our study showed an early pathological response to VO, which manifests in cardiomyocyte mechanical dysfunction and dysregulated signaling pathways associated with enhanced oxidative stress and an impaired protein quality control system.

## 1. Introduction

During the lifetime, and under both normal and pathological conditions, the heart continuously senses and responds to mechanical and metabolic stressors. Considering the very low regenerative capacity of the cardiomyocytes [1], they are challenged to sustain protein homeostasis via their protein quality control system (PQS) [2]. In general, PQS regulates protein folding and degradation through multilayered arrangement, which includes molecular chaperones, ubiquitin–proteasome system (UPS), and the autophagy-lysosome system [3]. Increasing evidence shows that dysfunctional PQS is involved in the development of various cardiac diseases including cardiac hypertrophy and heart failure [4,5].

Mechanical overload has been shown to induce cardiac remodeling and promote its transition to heart failure [6]. Volume overload (VO) generates mechanical stress to initiate stretch and remodeling of cardiac muscle [7,8]. However, chronic VO might initiate a maladaptive, compensatory response, followed by progressive hypertrophy and ventricular dilatation [7,8]. Aortic and mitral valves are the most frequently affected, leading to aortic valve and/or mitral annular calcification, which, in turn, causes either valve stenosis or regurgitation at an accelerated rate compared with the general population. In human valve insufficiencies (aortic and mitral valve), certain congenital abnormalities or chronic kidney diseases could lead to volume overload [9]. Moreover, mechanical stretching of cardiomyocytes was found to increase reactive oxygen species (ROS) production, which correlates with hypertrophic cardiac remodeling and apoptosis [10,11]. Indeed, we have previously provided evidence on the VO-induced hypertrophic remodeling in a rat model, which manifests in cardiomyocyte mechanical disturbances and contractile dysfunction mainly due to deranged phosphorylation status of myofilament proteins [12]. The latter is attributed to the increased oxidative state of kinases that are crucial for cardiomyocyte and heart function such as Ca^2+^/calmodulin-dependent protein kinase II (CaMKII) and protein kinase G (PKG) [12], indicating the potential contribution of oxidative stress to this modulation. Oxidative stress-induced impairments in signaling pathways [4], which can directly target myofilament proteins either via protein phosphorylation-based signaling pathways or via protein oxidation, lead to protein misfolding and aggregation [13,14]. The accumulation of oxidized, aberrant proteins can be detrimental for cell function, as it activates apoptotic cascades [15].

Molecular chaperons such as small heat shock proteins (sHSPs) serve as an endogenous first line of defense against protein misfolding [16]. When the misfolded protein cannot be rescued, sHSPs and their cochaperons mediate its clearance by directing the damaged protein bound to chaperon to autophagy/lysosomal and/or ubiquitin/proteasomal pathways [16]. Under various stress conditions, the expression level of HSPs was shown to be upregulated, thereby promoting cell survival [17]. In cardiomyocytes, and upon oxidative stress, the upregulated HSP27 and αβ-crystallin colocalize with titin at the Z-disc, thereby providing protection against Ig domain unfolding, aggregation, and subsequent high myocyte stiffness [18,19].

Both autophagy/lysosomal and the ubiquitin/proteasomal pathways represent a second line of defense against the damaged proteins that cannot be repaired [16,20]. The soluble, chaperone-bound misfolded protein can be ubiquitinated, i.e., marked for proteasomal degradation, or directly shuttled to the lysosomal degradation via HSP70 and its cochaperones [16]. Cellular autophagy can also be activated via insoluble, chaperon-bound aggregates that can be cleared in the lysosomes to some extent [16]. The remaining uncleared aggregates can initiate cardiac proteotoxicity. Although autophagy is highly involved in maintaining cardiomyocyte homeostasis [21,22], excessive autophagy was shown to exacerbate cardiac hypertrophy [23]. Hence, during cardiac stress such as hypertrophy and oxidative damage, an effective PQS is crucial for the prevention of cardiac proteotoxicity. However, PQS components and especially sHSPs can be injured by oxidative stress leading to the loss of their cytoprotective function [4,24,25]. The inadequate PQS results in a vicious cycle of protein aggregation, accumulation, and apoptosis [26]. Since myocardial VO-induced hypertrophic remodeling has been associated with oxidation of various kinases and increased cardiomyocyte stiffness in rats, we used this model to investigate the role of oxidative stress-induced PQS dysfunction.

## 2. Materials and Methods

### 2.1. Animals

The care and use of laboratory animals in the present study complies with the European Union directive (published as 2010/63/EU) and was approved by the Hungarian National Scientific Ethics Committee for Animal Experimentation and Animal Research Ethics Committee of the University of Szeged (approval ID: XXVIII./171/2018.; on 24 January 2018). Rats were housed in cages of the size recommended in the EU guidelines and were individually ventilated (Sealsafe IVC system, Techniplast S.p.a., Varese, Lombardy, Italy). Beneath the cages, the bedding material (Lignocell hygienic animal bedding) was changed at least two times per week [12]. The temperature-controlled (22 ± 2 °C) animal room had a 12 h light/dark cycle. The animals were fed standard rodent chow, and filtered tap water was available ad libitum.

### 2.2. Volume Overload-Induced Hypertrophy by Aortocaval Fistula

**Figure 1:**
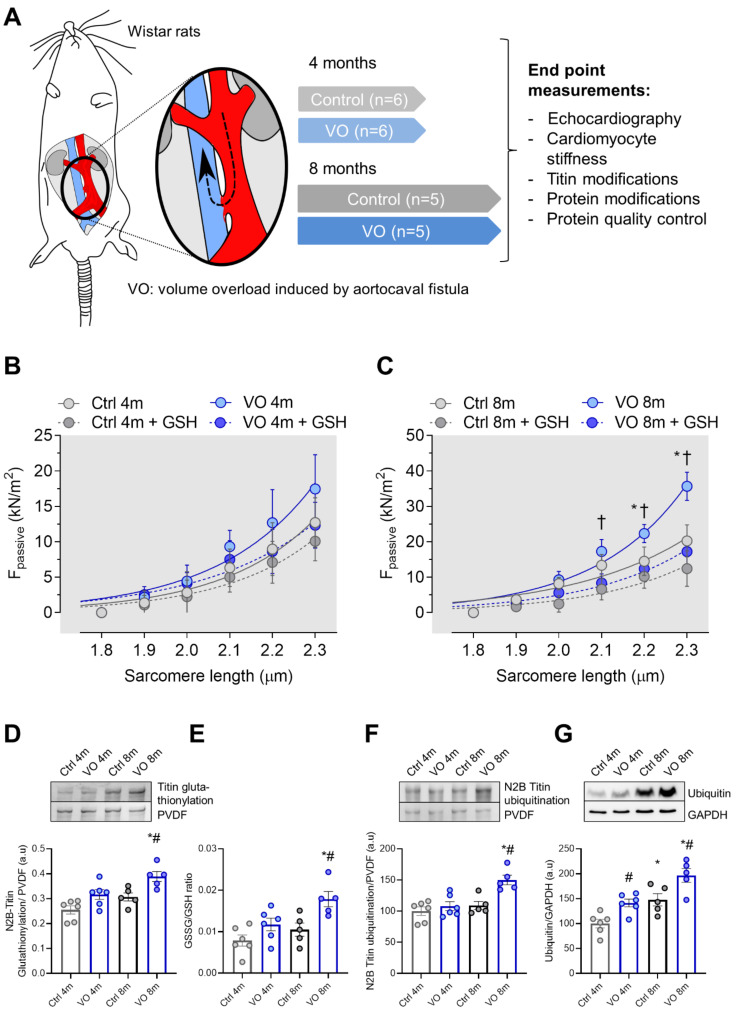
Protocol figure and titin oxidation and ubiquitination. (**A**) Experimental set-up, male Wistar rats were subjected to aortocaval fistula to develop volume overload-induced hypertrophy, (**B**) passive tension–sarcomere length relations of control (Ctrl) and volume overload (VO) cardiomyocytes in 4-month group with or without GSH treatment at sarcomere length (SL) 1.8–2.3 μm. (**C**) Passive tension–sarcomere length relations of control (Ctrl) and volume overload (VO) cardiomyocytes in 8-month group with or without GSH treatment at sarcomere length (SL) 1.8–2.3 μm. All force recordings were normalized for cardiomyocyte cross-sectional area. Curves represent third-order polynomial regressions. Data are shown as nonlinear regression fit of the means/medians; *n* = 5–6. * *p* < 0.05 Ctrl vs. VO 8-month, † *p* < 0.05; 8-month VO + GSH vs. 8-month VO, using one-way ANOVA Tukey multiple comparisons test. (**D**) S-glutathionylation of N2B-titin from left ventricular tissue. (**E**) GSSG/GSH ratio, (**F**) ubiquitination of titin, (**G**) ubiquitin level. Data are shown as mean ± SEM; *n* = 5–6. # *p* < 0.05 Ctrl vs. VO.; * *p* < 0.05 4-months vs. 8-months using one-way ANOVA Tukey multiple comparisons test.

As we previously described [12], two-month-old male Wistar rats were anesthetized with intraperitoneal (ip.) injection of pentobarbital sodium (Repose 50%, Le Vet. Pharma BV, Oudewater, Province of Utrecht, Netherlands). The animals were placed onto a heating pad in supine position, the abdomen was shaved, and the skin was disinfected with iodine solution. After a median laparotomy, the abdominal aorta was temporarily clipped between the left renal artery and the aortic bifurcation. An 18G needle was used to puncture the wall of the abdominal aorta into the wall of the inferior vena cava, creating a left-to-right shunt by an aortocaval fistula (Figure 1A). After removal of the needle, cyanoacrylate tissue glue (Loctite – Henkel KGaA, Dusseldorf, NRW, Germany) was applied to seal the surface of the aorta and to prevent bleeding. After surgery, the abdominal wall was closed in layers, and the animals received buprenorphine (0.05 mg/kg) subcutaneously. Every effort was made to minimize discomfort to the animals used in this study. Sham-operated animals were used as controls (Ctrl), in which the same procedure was performed but without the creation of an aortocaval fistula. For the development of volume overload-induced ventricular hypertrophy, animals were kept in standard housing conditions for 4 or 8 months (*n* = 5–6).

### 2.3. Transthoracic Echocardiography

As previously described [12], transthoracic echocardiography was performed at the end of 4 and 8 months using a commercial ultrasound machine (Vivid S5, GE Healthcare Hungary Ltd., Budapest, Pest, Hungary) equipped with second harmonic technology and a 10S 4–10.5 MHz-phased array sector probe. Left ventricular wall thicknesses, the diameter of the ventricular chamber, fractional shortening, and ejection fraction were measured from parasternal short-axis view. The diameter of abdominal aorta and vena cava inferior were measured from parasternal long-axis view using motion (M) mode images.

### 2.4. Termination of Animals

Rats were anesthetized by ip. injection of pentobarbital sodium (Repose 50%, Le Vet. Pharma BV, Oudewater, Province of Utrecht, The Netherlands) as previously described [12]. The right carotid artery was cannulated for measurement of blood pressure. Blood pressure and surface-lead electrocardiogram (ECG) were monitored for 10 min prior to termination (Haemosys data acquisition system, Experimetria Ltd, Budapest, Pest, Hungary). After recording hemodynamic parameters, the animals were sacrificed, and their hearts were isolated. The heart was dissected into left ventricle (LV), right ventricle (RV), and atria. Tissue samples were snap-frozen in liquid nitrogen and stored in an ultralow freezer at −80 °C until further measurements.

### 2.5. Passive Stiffness Measurement of Cardiac Sarcomere

Force measurements were performed on single demembranated cardiomyocytes (*n* = 10–13 cardiomyocyte/3–5 heart/group) as described before [27]. LV samples were thawed in relaxing solution (in mM: 1.0 free Mg^2+^; 100 KCl; 2.0 EGTA; 4.0 Mg-ATP; 10 imidazole; pH 7.0), mechanically disrupted, and incubated for 5 min in relaxing solution supplemented with 0.5% Triton X-100 (all from Sigma-Aldrich, St. Louis, MO, USA). The cell suspension was washed five times in relaxing solution. After selection of individual cardiomyocytes under an inverted microscope (Zeiss Axiovert 135, 40× objective; Carl Zeiss AG Corp, Oberkochen, BW, Germany), cardiomyocytes were fixed with shellac-dissolved in ethanol between a force transducer and a high-speed length controller (piezoelectric motor) using the Myostretcher system (Ionoptix, Westwood, MA, USA). The sarcomere length (SL) was monitored using a high-definition digital video camera, and the analysis software was provided by the manufacturer.

Ca^2+^-independent passive force (Fpassive) of cardiomyocyte in an SL range between 1.8 and 2.3 μm was measured in relaxing buffer at room temperature. Force values were normalized to myocyte cross-sectional area calculated from the cell diameter, assuming a circular shape. The forces were recorded at baseline and after antioxidant treatment, reduced glutathione (GSH) for 30 min (10 mM; Sigma-Aldrich – Merck KGaA, St. Louis, MO, USA), and interleukin-6 inhibitor (IL-6i, Siltuximab, 0.015 mL/L, EUSA Pharma Ltd, Hemel Hempstead, Hertfordshire, UK) for 45 min, respectively. All incubations were performed in relaxing solution.

### 2.6. Western Blot

LV tissue samples were solubilized in a modified Laemmli buffer (50 mM Tris-HCl at pH 6.8, 8 M urea, 2 M thiourea, 3% SDS *w*/*v*, 0.03% ServaBlue *w*/*v*, 10% *v*/*v* glycerol, 75 mM DTT, all from Sigma-Aldrich – Merck KGaA, St. Louis, MO, USA). Samples were heated at 96 °C for 3 min and centrifuged at 14,000 rpm for 3 min at 4 °C. From the supernatant, 25 µg of total protein/lane was loaded into 12% or 15% SDS gels and separated by electrophoresis. SDS-PAGE was performed at 90 V for 20 min followed by 120 V for 90 min. Proteins were then blotted to polyvinylidene difluoride (PVDF) membranes (Immobilon-P 0.45 μm; Merck Millipore, Burlington, MA, USA). Blots were blocked with 4% bovine serum albumin (BSA) in Tris-buffered saline with Tween (TBST; containing: 10 mM Tris-HCl; pH 7.6; 75 mM NaCl; 0.1% Tween; all from Sigma-Aldrich – Merck KGaA, St. Louis, MO, USA) for 1 h at room temperature (RT) and incubated with primary antibodies overnight at 4 °C (Table S1).

After washing with TBST, primary antibodies were detected with secondary goat antirabbit antibodies labeled with horseradish peroxidase (DakoCytomation; catalog number P0448 1:10,000) and enhanced chemiluminescence (ECL, Clarity Western ECL Substrate, BioRad Ltd, Hercules, CA, USA). Imaging was performed with ChemiDoc Imaging system (BioRad) and bands were quantified by densitometry using the Image Lab software (Version 6.1., Bio-Rad Ltd, Hercules, CA, USA) and Multi Gauge V3.2 software. To compare the protein load, GAPDH protein level (Sigma, 1:10,000) was evaluated for each sample. The obtained density values are expressed in arbitrary units (a.u).

### 2.7. Titin Gel Electrophoresis and Immunoblotting

To detect titin ubiquitination, LV samples were solubilized in a modified Laemmli buffer (50 mM Tris-HCl at pH 6.8, 8 M urea, 2 M thiourea, 3% SDS *w*/*v*, 0.03% ServaBlue *w*/*v*, 10% *v*/*v* glycerol, 75 mM DTT). For detecting titin oxidation, N-ethylmaleimide instead of DTT was used for solubilization. Samples were heated at 96 °C for 3 min, centrifuged for 3 min at 4 °C at 14,000 rpm, and then separated by agarose-strengthened 2% SDS-PAGE [28,29]. Gels were run at 2–4 mA constant current per gel for 16 h. Following SDS-PAGE, proteins were blotted onto PVDF membranes (Immobilon-P 0.45 μm; Merck Millipore, Burlington, MA, USA). Blots were preincubated with 4% bovine serum albumin in TBST for 1 h at RT followed by primary antibody incubation overnight at 4 °C.

Titin ubiquitination was investigated by HRP-conjugated secondary antirabbit antibodies (1:10,000), which were added the next day for 1 h at RT. After washing steps, blots were treated with ECL for developing chemiluminescence signal (see above). Chemiluminescence signals were normalized to signals obtained from Coomassie-stained PVDF membranes regarding the total amount of protein transferred. The results were quantified by densitometry using Multi Gauge V3.2 software (Fujifilm Ltd, Tokyo, Kanto, Japan).

### 2.8. Statistical Analysis

Data are expressed as mean ± SEM or as nonlinear regression fit the means/medians. All data were analyzed with one-way ANOVA followed by Tukey multiple comparison posthoc test. A significance value of *p* < 0.05 was chosen. * *p* < 0.05. 4-months vs. 8-months and # *p* < 0.05 Ctrl vs. VO. For force recordings, curves represent third-order polynomial regressions, and data are shown as nonlinear regression fit the means/medians; *n* = 5–6. * *p* < 0.05 Ctrl vs. VO 8-month, † *p* < 0.05, and 8-month VO vs. 8-month VO + treatment (GSH or IL-6i).

## 3. Results

### 3.1. Development of Cardiac Hypertrophy in VO Rats with Preserved Ejection Fraction

In our previous work [12], we reported increased heart/body weight ratio of VO animals and the development of cardiac hypertrophy with preserved ejection fraction (EF). Transthoracic echocardiography was performed at the end of 4 and 8 months. The EF was preserved and unchanged in all groups. At 8 months, the VO group showed increased left ventricular (LV) internal diameter during diastole (Figure S1A), not in systole (Figure S1B). Additionally, at 8 months, the VO group right ventricular (RV) internal diameter increased, as well as stroke volume and velocity-time index compared to their age-matched control group. There was no significant difference in mean arterial blood pressure (MABP, Figure S1C) among groups [12]. Additionally, the diameter of vena cava inferior was significantly increased in the 8-month VO group compared to control (Figure S1D), while fractional shortening (Figure S1E), LV posterior wall thickness (Figure S1F), and the diameter of abdominal aorta (Figure S1G) showed no changes between the groups.

### 3.2. Increased Titin-Based Myocardial Stiffness and Oxidation of the Giant Sarcomere Protein Titin

Increased cardiomyocyte stiffness is associated with cardiac hypertrophic remodeling. Titin’s mechanosensing function and its post-translational modifications are major determinants of cardiomyocyte stiffness. Therefore, we measured cardiomyocyte passive stiffness (Fpassive) in single-skinned cardiomyocytes within sarcomere length (SL) ranging between 1.8 and 2.3 μm before and after reduced glutathione (GSH) treatment. While the 4-month VO group showed no significant alterations in Fpassive as compared to the age-matched control group (Figure 1B) and no effect upon GSH treatment among the groups, Fpassive in the 8-month VO group was significantly elevated at SL 2.2 to 2.3 compared to the age-matched control group (Figure 1C). Increased Fpassive could be restored in 8-month VO cardiomyocytes after GSH treatment but remained unaltered in cardiomyocytes from the age-matched control group (Figure 1C), suggesting the contribution of protein oxidation to the elevated passive stiffness.

Since oxidative modifications of titin have been shown to modulate its stiffness, we aimed to evaluate the contribution of oxidation status (S-gluthationylation) of titin to the elevated cardiomyocyte stiffness. We found a significant increase in titin S-gluthationylation in the 8-month VO group as compared to the 8-month control (Figure 1D). The ratio of GSSG/GSH was significantly increased in the 8-month VO group compared to the 8-month control group (Figure 1E), indicating the imbalance of oxidant–antioxidant status in the VO model. Furthermore, titin ubiquitination was significantly elevated in the 8-month VO group compared to both the 4-month VO and 8-month control groups (Figure 1F). In addition, we have observed an increased total amount of ubiquitin over time between the 4-month vs. 8-month and VO vs. control groups, suggesting that the upregulation of ubiquitin levels are potentially VO- and age-dependent (Figure 1G).

### 3.3. Increased Oxidative Stress and Unaltered Inflammatory Markers in VO Animals

**Figure 2:**
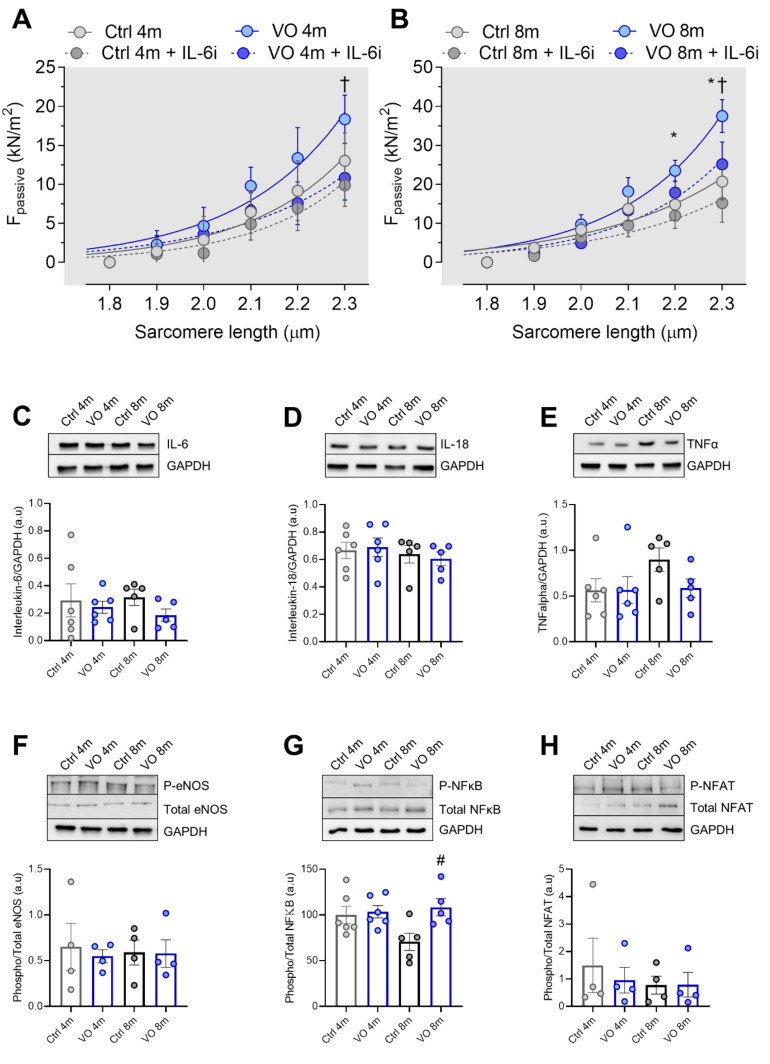
Inflammatory pathways. (**A**) Passive tension–sarcomere length relations of control (Ctrl) and volume overload (VO) cardiomyocytes in 4-month group with or without IL-6 inhibitor (IL-6i) treatment at sarcomere length (SL) 1.8–2.3 μm. (**B**) Passive tension–sarcomere length relations of control (Ctrl) and volume overload (VO) cardiomyocytes in 8-month group with or without IL-6 inhibitor treatment at sarcomere length (SL) 1.8–2.3 μm. All force recordings were normalized to cardiomyocyte cross-sectional area. Curves represent third-order polynomial regressions. Data are shown as nonlinear regression fit the means/medians; *n* = 5–6. * *p* < 0.05 Ctrl vs. VO 8-month, † *p* < 0.05; 8-month VO + IL-6i vs. 8-month VO, using one-way ANOVA Tukey multiple comparisons test. (**C**) Interleukin-6 protein level, (**D**) interleukin-18 protein level, (**E**) tumor necrosis factor alpha protein level, (**F**) phosphorylated/total endothelial nitrogen monoxide synthase (eNOS) protein ratio, (**G**) phosphorylated/total nuclear factor kappa B protein ratio, (**H**) phosphorylated/total nuclear factor of activated T-cells (NFAT) protein ratio. Data are shown as mean ± SEM; *n* = 4–6. * *p* < 0.05 4-months vs. 8-months, # *p* < 0.05 Ctrl vs. VO using one-way ANOVA Tukey multiple comparisons test.

Next, we assessed the contribution of the anti-inflammatory treatment to titin-based myocardial stiffness. In both the 4- and 8-month VO groups, interleukin-6 (IL-6) inhibition treatment decreased the elevated Fpassive; however, the reduction in Fpassive was significant only at high SL (2.3 µm) (Figure 2A,B). This was associated with unchanged protein levels of proinflammatory cytokines among all groups, including interleukin-6 (IL-6, Figure 2C), interleukin-18 (IL-18, Figure 2D), and tumor necrosis factor alpha (TNFα, Figure 2E). We also studied the phosphorylation status of downstream regulators of inflammation and stress signaling such as the endothelial nitric oxide synthase (e-NOS, Figure 2F), nuclear factor kappa B protein (NF-κB, Figure 2G), and nuclear factor of activated T-cells protein (NFAT, Figure 2H). None of them exhibited any alterations in their phosphorylation status except NF-κB (Figure 2G), which showed upregulated phosphorylation, indicating its activation in the 8-month VO group compared to the 8-month control group. Our findings suggest that changes observed in cardiomyocyte stiffness are independent of inflammation.

### 3.4. Alteration of Autophagy Markers in VO Animals

**Figure 3:**
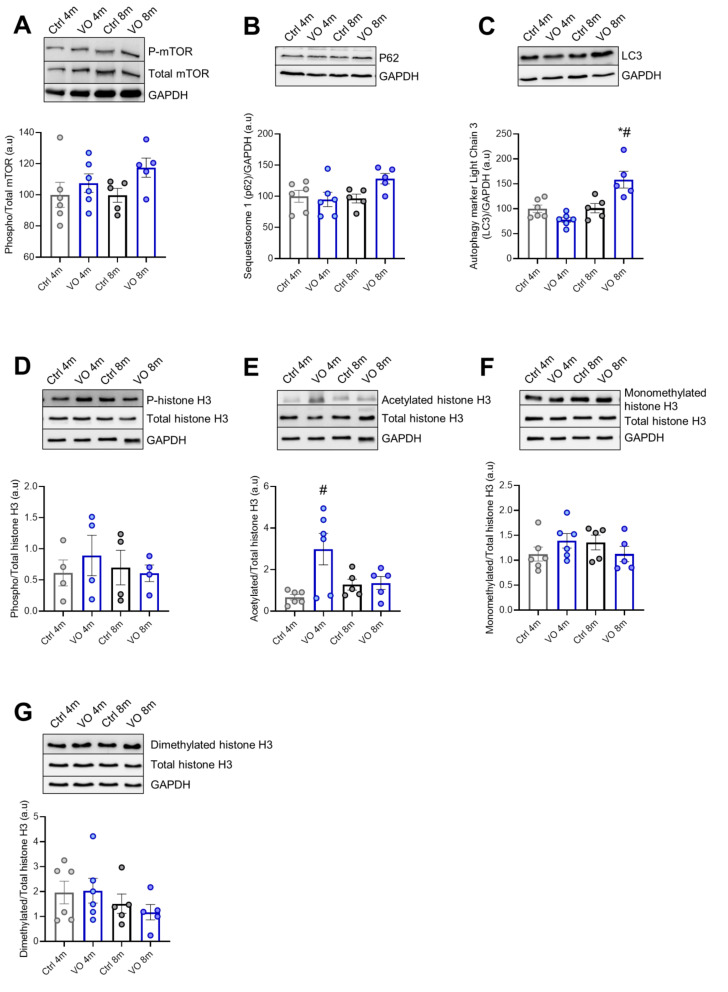
Downstream signaling pathway of cardiac hypertrophic growth and histone modifications. (**A**) Phosphorylated/total mTOR protein ratio, (**B**) sequestosome 1 (p62) protein level, (**C**) autophagy marker light chain 3 protein level, (**D**) phosphorylated/total histone 3 protein ratio, (**E**) acetylated/total histone 3 protein ratio, (**F**) monomethylated/total histone 3 protein ratio, (**G**) dimethylated/total histone 3 protein ratio. Data are shown as mean ± SEM; *n* = 4–6. * *p* < 0.05 4-months vs. 8-months, # *p* < 0.05 Ctrl vs. VO using one-way ANOVA Tukey multiple comparisons test.

We checked the mammalian target of rapamycin (mTOR), which is a regulator of several signaling pathways involved in autophagy, apoptosis, and cellular growth. The expression level of mTOR was unchanged among the groups; however, the 8-month VO showed an increasing tendency in mTOR phosphorylation compared to the age-matched control group (Figure 3A). Of note, phosphorylated/total protein ratio followed increasing trends in VO animals (Figure 3A) compared to control groups.

We assessed further downstream effectors that are known to play a major role in cellular autophagy such as sequestosome-1, also known as ubiquitin-binding protein p62, which is an autophagosome cargo protein. The expression level of p62 showed no significant differences between the groups (Figure 3B). On the other hand, the autophagy marker light chain 3 (LC3) was significantly upregulated in the 8-month VO group compared to the age-matched control group and the 4-month VO group as well (Figure 3C).

### 3.5. Histone’s Modifications due to VO

Epigenetics changes in cardiomyocytes, including histone post-translational modifications, have recently emerged as important players in the development of cardiac diseases such as hypertrophy and heart failure [30]. Therefore, we aimed to check for alterations in histone post-translational modifications in response to VO. Histone phosphorylation was unchanged among the groups (Figure 3D). Histone acetylation was significantly elevated in the 4-month VO group compared to the 4-month control. However, it declined in the 8-month VO group (Figure 3E). The methylation of histones was tested using antibodies against monomethylated (Figure 3F) and dimethylated histones (Figure 3G), resulting in no differences among the groups.

### 3.6. Apoptotic Markers and Caspases in VO Animals

**Figure 4:**
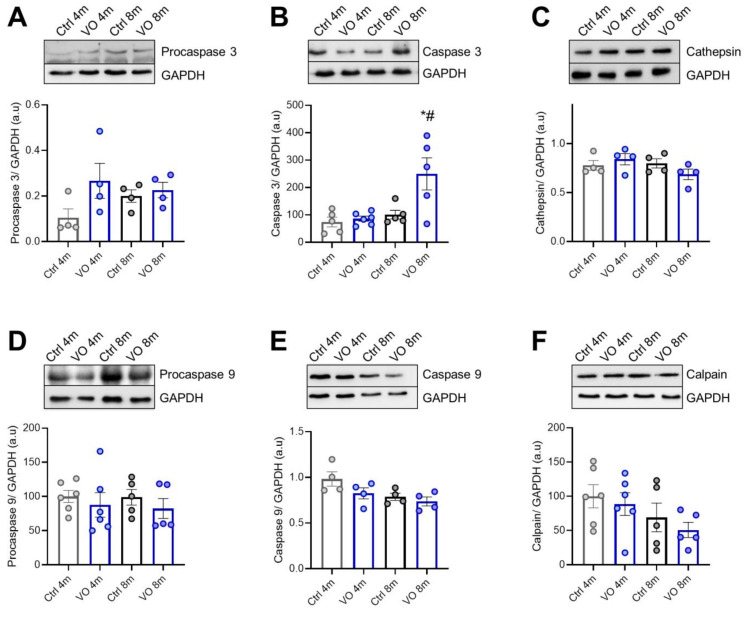
Proteases of the protein quality control system. (**A**) Procaspase 3 expression level from left ventricle tissue. (**B**) Active caspase 3 level, (**C**) cathepsin levels, (**D**) procaspase 9 expression level, (**E**) active caspase 9 level, (**F**) calpain levels. Data are shown as mean ± SEM; *n* = 4–6. * *p* < 0.05 4-months vs. 8-months, # *p* < 0.05 Ctrl vs. VO using one-way ANOVA Tukey multiple comparisons test.

We also evaluated cathepsin and caspases-mediated apoptosis in VO hearts. As shown in Figure 4A–F, active caspase 3 was significantly upregulated in the 8-month VO group vs. age-matched control group, while procaspase 3, procaspase 9, and active caspase 9 levels were unchanged. These results suggest a comparable caspase 9-dependent occurrence of apoptotic events in VO and control hearts. We then examined the changes in apoptotic signaling pathways in VO animals and hence determined the expression levels of proteases such as cathepsin (Figure 4C) and calpain (Figure 4F), which did not show any alteration among groups. However, calpain’s decreasing tendency could indicate that both age and VO may influence cardiac calpain levels.

### 3.7. Alterations in Heat Shock Proteins in VO Animals

**Figure 5:**
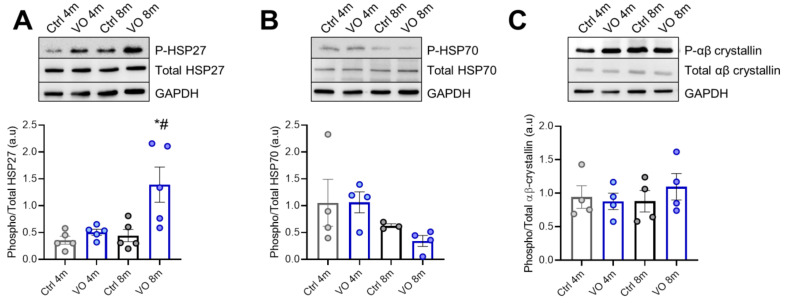
Chaperon proteins of the protein quality control system. (**A**) Phosphorylated/total heat shock protein 27 ratio, (**B**) phosphorylated/total heat shock protein 70 ratio, (**C**) phosphorylated/total αβ-crystallin ratio. Data are shown as mean ± SEM; *n* = 4–6. * *p* < 0.05 4-months vs. 8-months, # *p* < 0.05 Ctrl vs. VO using one-way ANOVA Tukey multiple comparisons test.

To evaluate the effect of VO and hypertrophic remodeling on PQS, we investigated the expression and phosphorylation levels of sHSPs. We found unchanged HSP27 expression levels among the groups; however, the phosphorylation level of HSP27 was significantly increased in the 8-month VO group compared to all other groups (Figure 5A). The expression level, as well as the phosphorylation status of HSP70, were slightly but nonsignificantly decreased over time, suggesting VO and age-dependent downregulation of HSP70-mediated proteostasis (Figure 5B). Both the expression and phosphorylation levels of αβ-crystallin were unchanged (Figure 5C).

## 4. Discussion

In our previous work and using a rat model which underwent aortocaval fistula to develop VO, we reported hypertrophic remodeling and cardiomyocyte mechanical disturbances as an early pathological response to VO [12]. Our data demonstrated elevated Ca^2+^ sensitivity and titin-based cardiomyocyte stiffness in the 8-month VO model, mainly due to CaMKII and PKG oxidation [12]. In this study, we aimed to unravel the molecular adaptations to VO that contribute to pathological remodeling such as oxidative stress, inflammation, impaired protein quality control system, histone modifications, and apoptosis.

Growing evidence from clinical and in vitro studies highlights the pivotal role of oxidative stress in the pathogenesis of cardiovascular diseases. Among the diverse pathological stimuli, mechanical strain induced by VO was shown to correlate with high levels of oxidative stress [31]. Due to the increased ROS production and the depressed antioxidant system, oxidative stress emerges as a central contributor to cardiomyocyte dysfunction [32]. One of the suggested mechanisms by which oxidative stress induces contractile dysfunction is the direct oxidative modifications of myofilament proteins and kinases [13]. Oxidative modifications could alter kinases affinity and/or accessibility, leading to an indirect modulation of signaling pathways and thereby causing cardiomyocyte dysfunction [13]. Previously, and in hypertrophic cardiomyopathy (HCM) patients, we provided evidence on the oxidative stress-related alterations in titin-based myocardial stiffness, as anticipated from the increased titin S-glutathionylation and ubiquitination compared to human–nonfailing control [4,24]. Furthermore, our 8-month VO rat model exhibited deranged phosphorylation status of myofilament proteins mainly due to kinases oxidation such as PKG and CaMKII [12]. In the current study, we found titin to be significantly S-glutathionylated and ubiquitinated, indicating the direct effect of oxidative stress-related protein modification. Of note, the observed modification of titin in the 8-months VO group refers potentially to an early stage of hypertrophic remodeling triggered by ROS. Consistently, the extent to which oxidative stress mediates cardiac dysfunction depends on the magnitude of ROS elevation [33]. While moderate levels of ROS induce fetal gene expression and hypertrophic remodeling, high ROS levels correlate with apoptotic phenotypes [34,35]. In addition, the 8-month VO group showed increasing tendencies toward titin glutathionylation, ubiquitination, and elevated myocyte stiffness compared to the 4-month VO group, indicating the progressive notion of oxidative stress-induced impairments. Considering the coexistence of oxidative stress and inflammation in various cardiac diseases, we investigated the ability of the IL-6 inhibitor to restore altered cardiomyocyte stiffness at higher sarcomere lengths and found comparable results upon the treatment. We also found the level of proinflammatory markers to be unchanged among the groups, indicating that the pathological response to VO was triggered by oxidative rather than inflammatory changes. However, it is plausible that VO correlated with high levels of NF-κB. It has been previously demonstrated that hypertrophic remodeling correlates with activation of NF-κB signaling mainly via ROS [36]. The upstream effectors of this pathway can be also activated via the VO-induced mechanical strain such as angiotensin II and endothelin-1 [37]. Despite the close association between oxidative stress and inflammation, it is unclear whether inflammation induces oxidative stress during the early hypertrophic remodeling response or vice versa. Onodi et al. found a lack of inflammasome (AIM2) activation, which points out that the activation of the inflammasome pathway is disease- and stage-specific [38]. However, when oxidative stress and inflammation chronically coexist, both processes are involved in a vicious cycle resulting in exacerbated pathological response and heart failure [39]. Clearly, altered oxidant/antioxidant state in the VO model in the absence of inflammation contribute to elevated cardiomyocyte stiffness and early hypertrophy.

PQS plays a fundamental role in the maintenance and the protection of long-lived cells such as cardiomyocytes. We have previously reported the involvement of dysfunctional PQS in HCM pathophysiology [4]. In this study, we checked the alterations in the mTOR signaling pathway, which is known to be involved in the regulation of hypertrophy, autophagy, and apoptosis [40]. mTOR phosphorylation was slightly increased in VO groups compared to matched controls. This was accompanied with the upregulation of degradation markers such as ubiquitin and LC3, indicating the activation of proteasomal and autophagy pathways in the 8-month VO group. Under normal and pathological conditions, the autophagy/lysosome system serves as a key regulator of protein homeostasis. However, upon stress, the elevated autophagosome synthesis and/or impaired autophagosome clearance can lead to the accumulation of autophagosomes and their cargo, which is associated with cell toxicity and death [41]. In the course of pressure-overload, downregulation of autophagy response is suggested to be involved in sarcomeric and mitochondrial insufficiency, leading to cardiac dysfunction and eventually heart failure [42]. On the other hand, excessive autophagy can be detrimental for cardiomyocyte function [23]. Oxidative stress-induced activation of autophagy was shown to contribute to the myocardial injury during ischemia/reperfusion in vivo [43]. Hence, the imbalanced autophagy response accounts for PQS dysfunction and cellular malfunction in cardiac diseases. Accordingly, the altered autophagy response in the 8-month VO group indicates the involvement of stress-induced PQS dysfunction in hypertrophic remodeling.

Autophagy flux is regulated by histone post-translational modifications. Both autophagy-related gene expression and histone modifications were suggested to constitute a regulatory feedback loop that can promote either the survival or apoptotic response upon autophagy induction [44]. Additionally, it has been demonstrated that chromatin remodeling is important, especially histone acetylation, in the control of gene expression in the heart. To better understand the VO-related alterations in PQS, we checked histone post-translational modifications in the VO group compared to the control group, including phosphorylation, acetylation, and methylation. We found an upregulation of histone acetylation in the 4-month VO group compared to the matched control group. Indeed, many proteins in the cell are acetylated and deacetylated, and similar to phosphorylation, acetylation of a protein can influence the stability of the protein, changing its enzymatic activity or facilitating new protein–protein interactions, thereby altering the cell function. Previous research reported the association between the elevated histone acetylation, excessive autophagy, and cell death [44]. In agreement, our results indicate a potential role of autophagy-related histone modifications in VO. Pharmacological suppression of histone deacetylases (HDACs) showed either blunt or amplified cell growth in cell culture models of cardiac hypertrophy, suggesting the potential of HDAC inhibitors as potential therapeutic agents in hypertrophic heart disease [45].

Considering the close association between dysregulated autophagy and apoptosis, we evaluated the caspase mediated apoptosis in VO and found upregulation of the active caspase 3 in the 8-month VO group compared to the matched control group. This result indicates the initiation of caspase 3-mediated apoptosis in response to VO. Furthermore, the levels of procaspase 9 and caspase 9 were unchanged among the groups, suggesting the induction of apoptosis by extrinsic rather than intrinsic pathway. The latter is promoted via the mitochondrial release of cytochrome C upon the induction by TNFα [46]. However, our model does not show any significant alterations in TNFα level among the groups, suggesting perhaps no severe mitochondrial damage with less leakage of cytochrome C in the early stage of the pathological response to VO, despite increased ROS level, as depicted from increased GSSG/GSH ratio. This indicates an activation of the extrinsic pathway and deactivation of intrinsic pathway due to upregulation of procaspase 3 and downregulation of procaspase 9, respectively [46]. Furthermore, we found no significant alterations in proteases’ protein levels among the groups. However, among the different sHSPs that we investigated, the phosphorylation level of HSP27 was significantly upregulated in the 8-month VO group compared to all other groups. In addition, αβ-crystallin phosphorylation was slightly upregulated in the 8-month VO group compared to the age-matched control. These findings demonstrate that sHSPs are activated at the early phase of VO, suggesting activation of sHSPs during cardiac stress and diseases. αβ-crystallin deficiency activates the nuclear factor of activated T cells (NFAT)/calcineurin pathway, consequently leading to cardiac hypertrophy development during no stress or minimal stress conditions, and worsens cardiac dysfunction in response to pressure overload [47]. Moreover, αβ-crystallin overexpression attenuates the hypertrophic response to pressure overload via suppression of the NFAT/calcineurin pathway [47]. Both HSP27 and αβ-crystallin are known to localize to the Z-disk and I-band regions of titin, thereby preventing Ig domains unfolding, aggregation, and subsequent elevation in titin-based myocardial stiffness [19,27,48,49,50]. However, previous research by us and others provided evidence on the oxidative stress-mediated disturbances in the cytoprotective function of sHSPs [4,24]. Despite the upregulation and activation of HSPs under oxidative conditions, they can directly be targeted by oxidative modifications, leading to the failure in their cytoprotective role. We have previously reported the oxidative stress-induced translocation of HSP27 away from the Z-disk and A-band in HCM [24]. In addition, sHSPs treatment reduced the elevated Fpassive in HCM cardiomyocyte [4]. Hence, the alterations in sHSPs activity in response to VO potentially indicate the oxidative stress-induced impairments in PQS.

## 5. Conclusions

We provided evidence on the disturbed cardiomyocyte function caused mainly by oxidative stress-mediated alterations in protein function and signaling pathways in the absence of inflammation in the chronic VO model. Mechanistically, we detected significant alterations in the fundamental components of PQS, as anticipated from the altered autophagy response, activated apoptosis, and altered phosphorylation status of sHSPs in VO. Taken together, here we describe the oxidative stress-mediated impairments in protein function and PQS as major contributors to hypertrophic remodeling in VO. Furthermore, our findings suggest, as potential clinical implications, the reduction of oxidative stress and restoration of PQS function as possible treatment approaches for the prevention of disease initiation and the transition toward heart failure.

## 6. Clinical Relevance and Future Therapeutic Approaches

Volume overload is a major risk factor for all-cause and cardiovascular mortality. Volume overload underlies hypertension, left ventricle hypertrophy and dysfunction, as well as pulmonary circulation overload in these patients. The results presented in this study suggest that oxidative stress, protein oxidation, and impaired PQS are potential mechanisms underlying the initiation and progression of the hypertrophy in VO. Under oxidative stress conditions, several regulators of signal transduction pathways are modified by high levels of reactive oxygen species. Cardiac dysfunction may also be attributed to impaired PQS. Failure of HSPs and their cochaperons in maintaining a balanced proteostasis might lead to the accumulation of toxic proteins and the induction of aggregation, apoptosis, fibrosis, and progression of cardiac dysfunction. Collectively, our data suggest that oxidative stress, but not inflammation, is a hallmark of VO-induced hypertrophy and could be a viable therapeutic approach to attenuating hypertrophy and cardiac dysfunction and preventing their progressions. One of the interventions that reduces oxidative stress and improves PQS is through compounds such as antioxidants that increase HSP expression [51]. However, clinical trials have shown that nonspecific antioxidative approaches, such as long-term vitamin supplementation, have failed to improve cardiac dysfunction in patients with cardiovascular events [52]. Taken together, strategies that target oxidative stress and impaired PQS may hold promise for novel drug discoveries.

## Data Availability

The data presented in this study are available in the article and supplementary materials.

## Supplementary Materials

The following supporting information can be downloaded at: https://www.mdpi.com/article/10.3390/antiox11112210/s1, Figure S1: Echocardiography data; Table S1: Primary antibody list.
