# Supplementary material for: Altered Cellular Protein Quality Control System Modulates Cardiomyocyte Function in Volume Overload-Induced Hypertrophy
Source: Antioxidants (Basel). 2022 Nov 8;11(11):2210. doi: 10.3390/antiox11112210 (PMC9686542; doi:10.3390/antiox11112210)
Supplement: Supplementary file 1 [file antioxidants-11-02210-s001.zip › antioxidants-1996036-supplementary.pdf]

Supplementary file for "Altered cellular protein quality control system modulates cardiomyocyte function in volume overload induced hypertrophy".

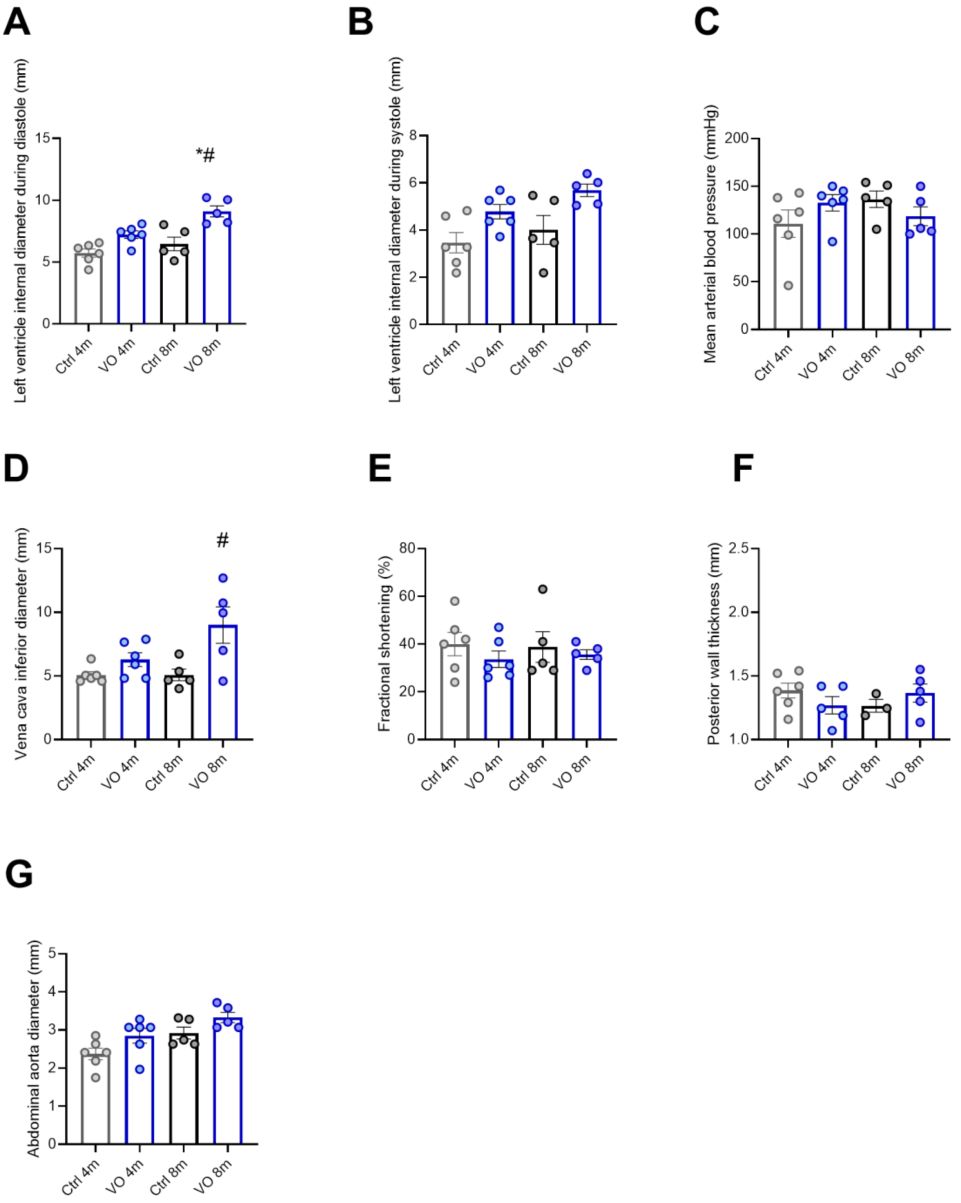

Figure S1. Echocardiography data. (A) left ventricular (LV) internal diameter during diastole (B) left ventricular (LV) internal diameter during systole (C) Mean arterial blood pressure (D) vena cava inferior diameter (E) fractional shortening, (F) left ventricle posterior wall thickness in/during diastole, (G) abdominalis aorta diameter. Data are shown as mean ± SEM. One-way ANOVA followed by Tukey multiple comparison post-hoc test. A significance value of $p < 0.05$ was chosen. *$p<0.05$. 4-months vs. 8-months and #$p<0.05$ Ctrl vs. VO.

Table S1. Primary antibody list

Antibody	Catalog number	Company	Dilution
HSP27	ab2790	Abcam	1:1000
Phospho HSP27	2406S	Cell signaling	1:1000
HSP70	ab2787	Abcam	1:1000

Phospho HSP70	PA536042	Invitrogen	1:1000
$\alpha\beta$-crystallin	ab13497	Abcam	1:1000
Phospho $\alpha\beta$-crystallin	ab5577	Abcam	1:1000
GAPDH	G9545	Sigma	1:10000
Titin glutathionylation	ab19534	Abcam	1:1000
Cathepsin L	sc32320	Santa Cruz	1:1000
Calpain	2556S	Cell signaling	1:1000
Pro-caspase 3	Sc7148	Santa Cruz	1:1000
Active caspase 3	Sc7148	Santa Cruz	1:1000
Pro-caspase 9	Sc7885	Santa Cruz	1:1000
Active caspase 9	Sc7885	Santa Cruz	1:1000
NFAT C2	PA579732	Invitrogen	1:1000
Phospho NFAT C2 (Ser 326)	PA5105754	Invitrogen	1:1000
eNOS	32027S	Cell signaling	1:1000
Phospho eNOS (Ser 1177)	9570S	Cell signaling	1:1000
IL-6	P620	Invitrogen	1:1000
IL-18	PA5-80719	Invitrogen	1:1000
TNFα	AMC3012	Invitrogen	1:1000
mTOR	2983S	Cell signaling	1:1000
Phospho mTOR	2971S	Cell signaling	1:1000
p62	39749S	Cell signaling	1:1000
LC3	12741S	Cell signaling	1:1000
ubiquitin	43124S	Cell signaling	1:1000
NF-κB	8242	Cell signaling	1:1000
Phospho NF-κB	3033	Cell signaling	1:1000
Total histone H3	PA5-31954	Invitrogen	1:1000
Phospho histone H3	PA5-17869	Invitrogen	1:1000
Acetlyted Lysine	9441S	Cell signaling	1:1000
Monomethylated histone H3	5928S	Cell signaling	1:1000
Dimethylated histone H3	2901P	Cell signaling	1:1000
